# Polymorphism of *ZBTB17* gene is associated with idiopathic dilated cardiomyopathy: a case control study in a Han Chinese population

**DOI:** 10.1186/2047-783X-18-10

**Published:** 2013-04-09

**Authors:** Xiaoping Li, Rong Luo, Xiaoyang Mo, Rongjian Jiang, Hong Kong, Wei Hua, Xiushan Wu

**Affiliations:** 1 Cardiac Arrhythmia Center, Cardiovascular Institute and Fu Wai Hospital, Chinese Academy of Medical Sciences, Peking Union Medical College, Beijing, 100037, China; 2 Department of Cardiology, Sichuan Academy of Medical Sciences and Sichuan Provincial People’s Hospital, Chengdu, Sichuan, China; 3 The Center of Heart Development, Key Lab of MOE for Development Biology and Protein Chemistry, College of Life Science, Hunan Normal University, Changsha, Hunan, 410081, PR China

**Keywords:** *actci*, Dilated cardiomyopathy, *hspb7*, Single nucleotide polymorphisms, *zbtb17*

## Abstract

**Background:**

Dilated cardiomyopathy (DCM) has been extensively investigated for many years, but its pathogenesis remains uncertain. The *ACTC1* gene was the first sarcomeric gene whose mutation was shown to cause DCM; recent studies have indicated that the *HSPB7* and *ZBTB17* genes are also associated with DCM. To assess the potential role of these three genes in DCM, we examined 11 single nucleotide polymorphisms (SNPs) in the *ZBTB17*, *HSPB7* and *ACTC1* genes: namely, rs10927875 in *ZBTB17*; rs1739843, rs7523558, and rs6660685 in *HSPB7;* rs533021, rs589759, rs1370154, rs2070664, rs3759834, rs525720 and rs670957 in *ACTC1.*

**Methods:**

A total of 97 DCM patients and 189 controls were included in the study. All SNPs were genotyped by matrix-assisted laser desorption/ionization time-of-flight mass spectrometry (MALDI-TOF-MS).

**Results:**

The genotype of SNP rs10927875 in *ZBTB17* (OR=5.19, 95% CI =1.00 to 27.03, *P*=0.05) was associated with DCM in a Han Chinese population. There was no difference in genotype or allele frequencies in *ACTC1* or *HSPB7* between DCM patients and control subjects.

**Conclusion:**

The *ZBTB17* polymorphism rs10927875 appears to play a role in the susceptibility of the Han Chinese population to DCM.

## Background

Dilated cardiomyopathy (DCM) is a primary myocardial disease and genetically heterogeneous disorder. It is characterized by progressive systolic dysfunction due to cardiac chamber dilatation and inefficient myocardial contractility. The phenotype in idiopathic DCM is characterized by cardiac muscle dysfunction in the absence of secondary causes. A majority of DCM cases are sporadic, but familial transmissions are observed in 25% to 35% of cases and are inherited as autosomal dominant, recessive, or X-linked traits with variable expressivity and penetrance [1]. Studies have led to the identification of mutations in more than 15 different genes coding for sarcomeric, cytoskeletal, or regulatory proteins as a primary cause of DCM [2,3].

*HSPB7* encodes a cardiovascular small heat shock protein, HSP70, which belongs to the small HSP (sHSP) family [4]. *HSPB7* is also called cardiovascular HSP, because of its selective expression in cardiovascular tissues [5]. In general, the expression and activation of heat shock proteins are influenced by elevated temperatures as well as ischemia, hypoxia and acute cellular stress [6,7]. In human beings, genetic variants in *HSPB7* have been reported to be associated with advanced heart failure and systolic dysfunction of unspecific origin [8,9]. The single nucleotide polymorphism (SNP) is the most common type of genetic variation in the human genome, and recent DCM study has used large-scale screening on SNPs in European populations to show that *HSPB7* gene SNPs (rs1739843) are associated with DCM [10].

The *ACTC1* gene encodes skeletal muscle α-actin, which is the predominant actin isoform in the sarcomeric thin filaments of adult skeletal muscle; α-actin is also essential for cardiac muscle contraction. Each myosin head interacts with two adjacent actin monomers along the cardiac filament structure. The *ACTC1* gene was the first sarcomeric gene whose mutation was shown to cause DCM [11]. To our knowledge, however, there have been no reports on the association between *ACTC1* SNPs and DCM.

The *ZBTB17* gene encodes protein 17, which contains both zincfinger and BTB domains. Protein 17 is also known as myc-interacting protein 1 (MIZ-1) and is a transcription factor of 87 kDa containing 13 zinc finger domains at its carboxy-terminal end and a BTB/POZ domain at its N-terminus [12]. MIZ-1 was originally identified as an interacting partner of the c-Myc proto-oncogene [12] and, depending on its interacting partner, MIZ-1, it can either activate or repress the transcription of its target genes [13-16]. Recently, a GWAS (genome-wide association study) study on DCM indicated that the *ZBTB17* gene SNP rs10927875 was associated with DCM [17].

Based on these findings, we hypothesized that some DCM incidences in patients are associated with certain polymorphisms of *ZBTB17, HSPB7* and *ACTC1* genes. To our knowledge, there is no interaction among the three genes. To test this hypothesis, we used matrix-assisted laser desorption/ionization time-of-flight mass spectrometry (MALDI-TOF-MS) to investigate 11 SNPs in *ZBTB17, HSPB7* and *ACTC1* in both DCM patients and normal subjects from a Han Chinese population.

## Methods

### Subjects

This case control study enrolled 97 unrelated DCM patients from the Fuwai Hospital in northern China from January 2006 to January 2007. Clinical diagnoses were made in accordance with the revised criteria [18], in which primary DCM was defined as systolic dysfunction (left ventricular (LV) ejection fraction <50%) with LV dilation in the absence of an apparent secondary cause of cardiomyopathy. In addition, 189 healthy unrelated individuals, without any sign or history of cardiovascular disease, were enrolled from a routine health survey as controls in this study. Patients with a history of hypertension, coronary heart disease, cardiac valve disease, diabetes, acute viral myocarditis, systemic diseases of putative autoimmune origin and a family history of DCM were intentionally excluded. This study was approved by the ethics committee of Fuwai Hospital; the subjects involved were from the Han nation in northern China. All subjects involved were aware of the study and gave written informed consent.

### Isolation of DNA and genotyping by MALDI-TOF-MS

Blood samples were collected from patients using tubes containing ethylenediaminetetraacetic acid (EDTA). Genomic DNA was isolated from whole blood, and genotyping was performed by MALDI-TOF-MS, as described previously [19]. SNP genotyping was performed using the MassARRAY system (Sequenom, San Diego, California) using the MALDI-TOF-MS method. Completed genotyping reactions were spotted onto a 384-well spectroCHIP (Sequenom) using a MassARRAY Nanodispenser (Sequenom), and the genotype was determined by MALDI-TOF-MS. Genotype calling was performed in real time with MassARRAY RT software version 3.1 (Sequenom) and analyzed using the MassARRAY Typer software version 4.0 (Sequenom) (Table 1).

**Table 1:** **Sequences of PCR primers, amplicon sizes, temperatures and GC content in SNPs of *ZBTB17, HSPB7 *, and *ACTC1 *in DCM patients and control subjects**

**Markers**	**Forward primer (5**^**′**^**-3**^**′**^**)**	**Reverse primer (5**^**′**^**-3**^**′**^**)**	**Amplicon size (bp)**	**Temperature (°C)**	**GC (%)**
***ZBTB17***					
rs10927875	GCCAGAGTGGATGATCACTG	TTCTGTTTCCACCACTGTAG	91	54.2	55
***HSPB7***					
rs1739843	TGTCCTCACTCTGCCATCAC	TGGGCAGAGGGAGCCTGAG	99	50.8	50
rs7523558	GCCCAGCACCTATTTATAGC	CTCATAGGCCAGTGATGAAG	94	46.6	35
rs6660685	TACTGTCCCACAGCCAGCAC	TCTTCTCAGCCTCCTGGTG	98	50.8	58.8
***ACTC1***					
rs533021	GGCCTTCCATTTGAATATGC	GTATGAGAGCACATTTTCTG	96	45.1	33.3
rs589759	CTTCTCACCTCCCTAATTCC	TGTGGCCTGGAGGCTTTAAG	96	49.4	45
rs1370154	GCAGCAACTCATTCTAGAG	TTTCATACCTGAAAAGCAG	108	45.2	24
rs2070664	CATAACAATGACTGCTGCAC	ATAGCTTGTGGAGATAGGTC	100	46.9	60
rs3759834	TCTCCATCAAAGTATTTGCC	GATGAGCATCTTTAAACTGG	100	52	45
rs525720	GTTCTTGACTGGAGCTTTG	GCCAGTGCCCAGTTTTCTAT	103	45.6	33.3
rs670957	ATGCCAAGAGTAGAACTGCC	TCTGACACCAGTCCCATTTC	97	45.5	50

### Statistical analyses

Differences in the distributions of demographic characteristics, selected variables, and genotypes of the *ZBTB17*, *HSPB7*, and *ACTC1* gene variants between the cases and controls were evaluated using the chi-square (*χ*^2^) test; continuous variables were analyzed by an unpaired Student’s *t* test. Associations between Z*BTB17*, *HSPB7*, and *ACTC1* genotypes and the risk of DCM were estimated by computing the odds ratios (ORs), and their 95% confidence intervals (CIs) using logistic regression analysis. Hardy-Weinberg equilibrium was tested by a goodness-of fit χ^2^ test to compare the observed genotype frequencies with those expected among control subjects. All statistical analyses were performed with SPSS 16.0 (IBM; Armonk, USA), and a *P* value ≤0.05 was considered statistically significant.

## Results

The study included 286 subjects, with 97 patients with DCM and 189 healthy control subjects. The mean age of control subjects was 54.0±3.6 years; 79.4% were men. Patients with DCM were of similar age (51.6±12.0 years), and there was a similar percentage of men (77.3%) (*t*=1.700, *P*=0.092 and *χ*^2^ =0.160, *P*=0.689, respectively). The percentage of smokers among DCM patients was 43.3%, but no data on smoking status were collected from healthy subjects. No subjects in the present study were pregnant or peripartum. Dilated cardiomyopathy was diagnosed as systolic dysfunction (LV ejection fraction <50%) with LV dilation detected by echocardiogram in the absence of an apparent secondary cause of cardiomyopathy. In DCM patients, the mean LV ejection fraction was 32.0±8.4%, the LV diameter was 67.7±8.6 mm, and the left atrium diameter 42.7±7.6 mm. The SNP genotype calling was performed in real time with the Mass ARRAY system and MALDI-TOF-MS. A few patients and controls whose genotypes could not be called: their genotypic data were omitted from analysis,thus the total number of called genotypes for some SNPs totaled less than 286. The observed and expected genotype frequencies of each SNP were not significantly different between DCM patients and control subjects, indicating that the samples fit the assumption of Hardy-Weinberg equilibrium (Table 2).

**Table 2:** **DNA variants identified in*ZBTB17*,*HSPB7*, and*ACTC1***

**Markers**	**Location of nucleotide change**	**Amino acid change**	**Note**	**ObsHET**	**PretHET**	**HWE(*P*)**
***ZBTB17***						
rs10927875	Intron 16299312 C>T	Non-coding	Nor-reported non-coding SNP	0.025	0.025	0.9417
***HSPB7***						
rs1739843	Intron 16343254 C>T	Non-coding	Reported non-coding SNP	0.413	0.381	0.7898
rs7523558	5^′^ near gene 16346732 G>A	Non-coding	Nor-reported non-coding SNP	0.156	0.15	0.7933
rs6660685	5^′^ near gene 16346988 G>A	Non-coding	Nor-reported non-coding SNP	0.029	0.028	0.9112
***ACTC1***						
rs533021	3^′^ UTR 35080931 T>C	Non-coding	Reported non-coding SNP	0.446	0.453	1.0000
rs589759	3^′^ UTR 35081574 C>T	Non-coding	Reported non-coding SNP	0.524	0.497	0.2017
rs1370154	3^′^ UTR 35082225 G>A	Non-coding	Reported non-coding SNP	0.45	0.466	0.8150
rs2070664	Intron 35085201 A>G	Non-coding	Nor-reported non-coding SNP	0.47	0.491	0.9324
rs3759834	5^′^ near gene 35088705 T>C	Non-coding	Nor-reported non-coding SNP	0.06	0.058	0.6522
rs525720	5^′^ near gene 35089134 G>A	Non-coding	Nor-reported non-coding SNP	0.254	0.248	0.4169
rs670957	5^′^ near gene 35089432 A>G	Non-coding	Nor-reported non-coding SNP	0.482	0.5	0.5963

HWE(*P*), *P* value from Hardy-Weinberg equilibrium test in the control group; ObsHET, observed heterozygosity; PretHET, present heterozygosity.

Using the *χ*^2^ test, we compared genotypes and allele frequencies between cases and controls. Our results showed that the minor T allele frequency of SNP rs10927875 in *ZBTB17* (OR=5.19, 95% CI =1.00 to 27.03, *P*=0.05) was associated with DCM in the Chinese population. In addition, as the number of homozygote ‘TT’ genotype carriers was zero among patients and controls and the minor T allele was considered a risky exposure factor, we compared the frequency of the heterozygous ‘CT’ genotype and the homozygous ‘CC’ genotype. The results showed that the ‘CT’ genotype might also be associated with DCM (OR=5.31, 95% CI =1.01 to 27.92, *P*=0.049). Multivariate analysis (logistic regression analysis) was also used to estimate the association of the T allele in rs10927875 and DCM. Logistic regression analysis indicated that the T allele was an independent risk factor for DCM after excluding some confounding factors, such as age and sex (OR= 6.42, 95% CI: 1.19 to 34.77; *P*= 0.031).

There was no difference in genotype or allele frequencies in *ACTC1* or *HSPB7* between DCM patients and control subjects. The allelic and genotypic frequencies of SNPs in cases and controls and the statistical analysis results are shown in Table 3.

**Table 3:** **Genotypic and allelic frequencies of SNPs from*ZBTB17, HSPB7*, and *ACTC1 *genes in DCM patients and controls**

**Marker**	**Genotype**			**Allele**		***P *value**	**OR(95% CI)**
***ZBTB17***							
rs10927875	C/C	C/T	TT	C	T	0.05	5.19(1.00, 27.03)
Patients	88(0.946)	5(0.054)	0	181(0.973)	5(0.054)		
Controls	187(0.989)	2(0.011)	0	376(0.995)	2(0.011)		
***HSPB7***							
rs1739843	C/C	C/T	T/T	C	T	0.81	0.95(0.64, 1.42)
Patients	49(0.521)	43(0.457)	2(0.021)	141(0.75)	47(0.250)		
Controls	103(0.545)	74(0.392)	12(0.063)	280(0.741)	98(0.259)		
rs7523558	A/A	A/G	G/G	A	G	0.96	0.98(0.52, 1.87 )
Patients	0(0)	15(0.161)	78(0.839)	15(0.081)	171(0.919)		
Controls	1(0.005)	29(0.153)	159(0.841)	31(0.082)	347(0.918)		
rs6660685	A/A	A/G	G/G	A	G	0.11	3.29(0.78, 13.91)
Patients	0	5(0.053)	90(0.947)	5(0.026)	185(0.974)		
Controls	0	3(0.016)	181(0.984)	3(0.008)	365(0.992)		
***ACTC1***							
rs533021	C/C	C/T	T/T	C	T	0.36	1.19(0.82, 1.73)
Patients	13(0.149)	39(0.448)	35(0.402)	65(0.374)	109(0.626)		
Controls	21(0.111)	84(0.444)	84(0.444)	126(0.333)	252(0.667)		
rs589759	C/C	C/T	T/T	C	T	0.25	1.22(0.87, 1.73)
Patients	25(0.258)	48(0.495)	24(0.247)	98(0.505)	96(0.495)		
Controls	54(0.286)	102(0.540)	33(0.175)	210(0.556)	168(0.444)		
rs1370154	A/A	A/G	G/G	A	G	0.77	0.93(0.58, 1.50)
Patients	7(0.156)	18(0.4)	20(0.444)	32(0.356)	58(0.644)		
Controls	26(0.141)	85(0.462)	73(0.397)	137(0.372)	231(0.628)		
rs2070664	A/A	A/G	G/G	A	G	0.84	1.04(0.73, 1.47)
Patients	33(0.344)	42(0.438)	21(0.219)	108(0.562)	84(0.438)		
Controls	62(0.328)	92(0.487)	35(0.185)	216(0.571)	162(0.429)		
rs3759834	C/C	C/T	T/T	C	T	0.72	0.82(0.29, 2.37)
Patients	0	5(0.053)	90(0.947)	5(0.026)	185(0.974)		
Controls	0	12(0.063)	177(0.937)	12(0.032)	366(0.968)		
rs525720	A/A	A/G	G/G	A	G	0.42	0.81(0.48, 1.36)
Patients	2(0.022)	19(0.211)	69(0.767)	23(0.128)	157(0.872)		
Controls	3(0.016)	52(0.275)	134(0.709)	58(0.153)	320(0.847)		
rs670957	A/A	A/G	G/G	A	G	0.09	1.36(0.95, 1.94)
Patients	19(0.211)	44(0.489)	27(0.3)	82(0.456)	98(0.544)		
Controls	55(0.293)	90(0.479)	43(0.229)	200(0.532)	176(0.468)		

CI, confidence interval; OR, odds ratio in which the minor allele was viewed as an exposure factor in the case-controlled study.

Because some studies have documented that men account for a much larger percentage of DCM patients [18,20], we compared the frequencies of the genotype of SNP rs10927875 between cases and controls within sexes. For both men and women, the genotype of SNP rs10927875 showed no significant difference between patients and control subjects (*χ*^2^=3.374, *P*=0.066; *χ*^2^=1.889, *P*=0.169).

**Figure 1:**
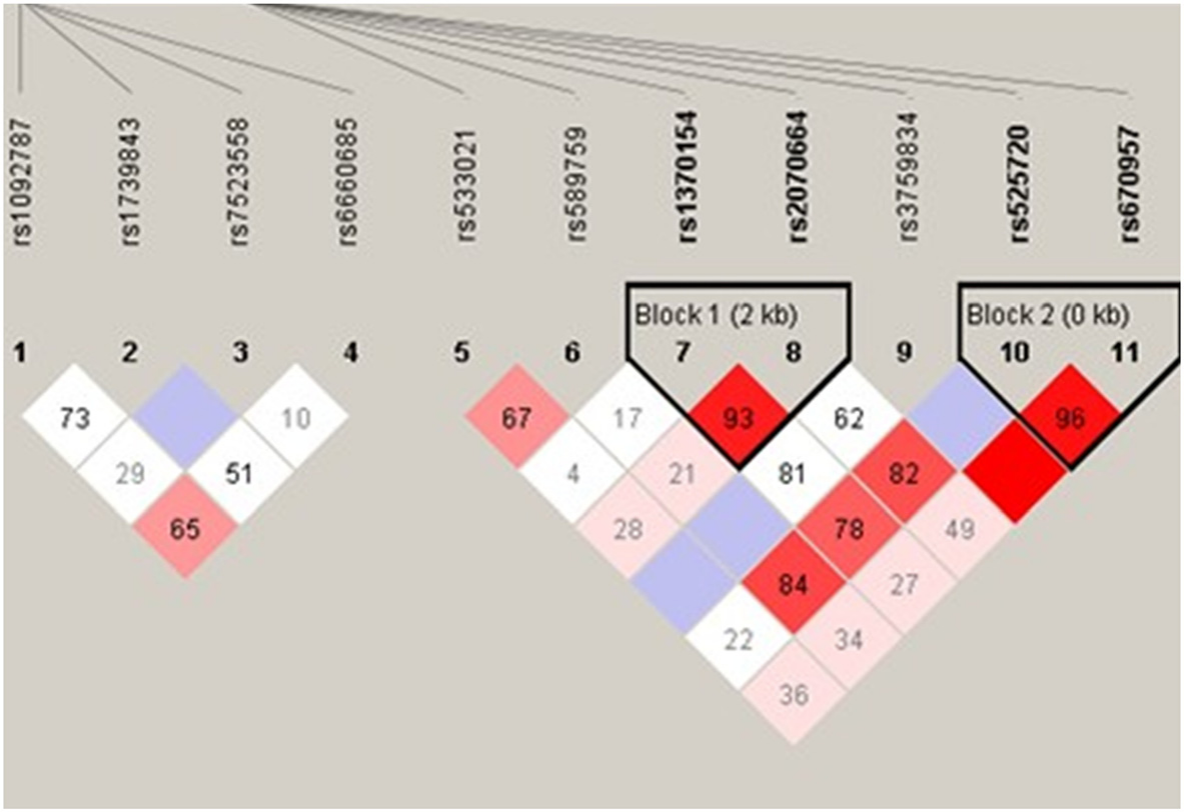
**Pairwise linkage disequilibrium (LD) values calculated between tagging SNPs in *ZBTB17, HSPB7*, and *ACTC1. ***The value within each diamond represents the pairwise correlation between tagging SNPs (measured as *D*^*′*^), defined by the upper left and the upper right sides of the diamond. The diamond without a number corresponds to *D*^*′*^= 1. Shading represents the magnitude and significance of the pairwise LD, with darker red reflecting higher LD values and white indicating lower LD values.

Non-random associations between polymorphic variants at different loci on the three genes were then measured by the degree of linkage disequilibrium (LD). Analysis of LD showed that none of the SNPs in the three genes was in high LD in the DCM patients (Figure 1, *D*^*′*^<0.80). Haplotype analysis showed that GG, AA, GA, and AG in block 1 and GG, GA, and AA in block 2 were not correlated significantly with DCM (Figure 1, Table 4).

**Table 4:** Haplotype analysis of SNPs between DCM patients and control subjects

	**Haplotype**	**Frequency (DCM patients)**	**Frequency (control subjects)**	***χ***^**2**^	***P*value**
**Block 1**	GG	0.426	0.418	0.028	0.8681
	AA	0.371	0.363	0.038	0.8456
	GA	0.192	0.209	0.211	0.6460
	AG	0.011	0.010	0.008	0.9293
**Block 2**	GG	0.544	0.464	3.166	0.0752
	GA	0.328	0.383	1.589	0.2075
	AA	0.127	0.150	0.524	0.4691

## Discussion

We have analyzed the relationship between DCM and SNPs in the genes *HSPB7*, *ZBTB17*, and *ACTC1*. It has been shown that the T allele of *ZBTB17* (rs10927875) and the ‘CT’ genotype are associated with DCM in a subgroup of patients within the Chinese population. Moreover, the T allele in rs10927875 was determined to be an independent risk factor for DCM, suggesting that *ZBTB17* polymorphisms (rs10927875) play an important role in the susceptibility to DCM in the Chinese population.

Dilated cardiomyopathy is a common form of heart muscle disease with a prevalence of 1/2,500 in the general population. It represents a major cause of cardiovascular morbidity and mortality and is characterized by systolic dysfunction, dilation, and impaired contraction of the ventricles, often leading to chronic heart failure and eventually requiring cardiac transplantation [18]. In approximately 35% of cases, DCM is a familial disease [20]. Mutations in both sarcomeric and cytoskeletal genes have been implicated in DCM, but the variable expression and penetrance of each gene that harbors a different mutation result in vast clinical heterogeneity among patients. Knowledge of the genetic risk factors for DCM is important to initiate treatment prior to symptomatic onset of the disease to delay its occurrence or possibly halt its progression. To date, only a few common susceptibility alleles for sporadic DCM have been identified from candidate-gene approaches [20,21].

The most common form of genetic variation is the SNP, defined according to the variation of a single nucleotide occurring in more than 1% of the population. The majority of SNPs are likely to be allelic variants that do not affect the expression or function of a protein. Such SNPs are commonly used as genetic markers to localize nearby disease-causing variations in linkage and association analyses. Single nucleotide polymorphisms that directly influence phenotype may be located within coding or regulatory regions of genes and can result in disease. In contrast, SNPs within regulatory regions tend to have more quantitative effects; for example, they may alter the expression level of a receptor or signaling protein, resulting in a more subtle variation in the associated phenotype [22].

Understanding the genetic heterogeneity of complex polygenic diseases like DCM is challenging. One of the many achievements in medical genetics over the past decades has been the ability to visualize sequence differences directly in DNA. These differences, or polymorphisms, serve as genetic markers of disease [23]. Recently, a GWAS was performed on a sporadic form of DCM in a European population. Another study screened approximately 2,000 candidate genes previously implicated in cardiovascular disease in more than 1,900 sporadic DCM cases in German and French populations. These studies showed that the SNPs rs10927875 and rs1739843 in *ZBTB17* and *HSPB7*, respectively, were associated with DCM [10,17].

rs10927875 is located in an intron of *ZBTB17* on chromosome 1p36.2-p36.1. The locus of interest covers approximately 210 kb in a genomic region; exhibits strong LD; and spans several other genes, including *SPEN* (spen homolog, transcriptional regulator), *HSPB7*, *CLCNKA* (chloride channel Ka), and *CLCNKB* (chloride channel Kb) [17]. Our data, together with the results from previous GWAS on DCM, substantiate the importance of rs10927875 and related polymorphisms in the *ZBTB17* locus for DCM susceptibility [17]. However, the biological mechanism explaining the association between the polymorphism rs10927875 and DCM risk remains unclear. Preliminary data suggest that MIZ-1 (a transcription factor encoded by *ZBTB17*) may play a role in the transcriptional activation of numerous other genes [13-16]. MIZ-1 is composed of 13 zinc finger domains at its C terminus and a BTB/POZ (Broad-complex, Tramtrack, and Bric-a-brac/pox virus zinc finger) domain at its N-terminus [13]. Whether MIZ-1 activates or represses the transcription of its target genes depends on its interacting partner. The genes that encode the negative cell cycle regulators Cdkn2b [13,16] or Cdkn1a [24,25] have been validated as direct MIZ-1 targets, and c-Myc has been shown to be recruited to the Cdkn1a promoter by MIZ-1; this interaction blocks Cdkn1a induction by p53 and other activators in cancer cells [13,16,24,25]. As a result of Miz-1 actions, c-Myc switches the cell fate from cell cycle arrest to apoptosis in response to p53-dependent activation [24]. *In vitro* studies have shown that the MIZ-1 pathway is indispensable for the ability of c-Myc to act as an oncogene, inducing both cell cycle progression and transformation. The ability of c-Myc to bind and inactivate MIZ-1 is required for c-Myc’s induction of apoptosis in growth factor-deprived primary human fibroblasts [26]. In a recent study, MIZ-1 was shown to have an additional function in pre-T cell differentiation at the b-selection stage of DN3 cells, the first critical checkpoint in the maturation of pre-T cells [27]. As it is well known that both immunity and apoptosis play an important role in the pathology of DCM, the polymorphism in the *ZBTB17* gene might be associated with DCM. Clearly, functional studies are required to support these hypotheses.

It has become clear that certain heat shock proteins functioning as molecular chaperones form a potent natural defense against proteotoxic stress induced by protein misfolding diseases. *HSPB7* is known to be expressed in cardiovascular and insulin-sensitive tissues [5]. In general, the expression and activation of heat shock proteins are influenced by elevated temperatures as well as ischemia, hypoxia and acute cellular stress [6,7]. In aging skeletal muscle, an increase in HSP70 protein content has been observed [28]. Recently, Cappola reported an association between rs1739843 and heart failure [9]. The findings on *HSPB7* are also in line with a previously reported large-scale resequencing approach of four biologically relevant cardiac signaling genes, in which *HSPB7* sequence diversity was detected in sporadic cardiomyopathy [8,10]. However, in the present case control study, we identified a polymorphism (rs1739843) in intron 2 of the *HSPB7* gene that had no association with susceptibility to DCM in a Han Chinese population. Many factors account for the difference between the results of our study with the abovementioned studies: the subjects differed in race, the enrolment criteria for DCM patients differed, the patient statuses differed, and so on. In addition, we did not find any association between SNPs in *ACTC1* and DCM.

To our knowledge, this study is the first to describe the association of SNPs in *ZBTB17*, *HSPB7*, and *ACTC1* with DCM in a Han Chinese population. This study has provided the first evidence that a *ZBTB17* gene SNP (rs10927875) is a risk factor for susceptibility to DCM in Chinese populations. The present study found no association between SNPs in HSPB7 and ACTC1 genes with DCM.

## Conclusion

In conclusion, we have provided the first evidence that a *ZBTB17* gene SNP (rs10927875) is a risk factor for susceptibility to DCM in Chinese populations. However, genetic polymorphisms vary greatly among different ethnic populations; therefore, further studies in other populations are needed to exclude a population-oriented association.

### Limitations

Some limitations may have affected the accuracy of the results. One limitation was the relatively small sample size. In addition, as there were a few patients and controls whose genotypes could not be called, their genotypic data were omitted from analysis, and thus, the total number of called genotypes for some SNPs was less than 286. Secondly, no data on body mass index, smoking status, or pregnancy history were obtained from healthy subjects. Thirdly, as the control subjects were recruited using a routine health survey and lacked any signs or history of cardiovascular disease, we did not obtain echocardiograph parameters to fully exclude cardiovascular diseases in the healthy subjects. Last but not least, a possible limitation of our study is the lack of a more comprehensive genetic analysis of other SNPs in *ZBTB17*, which may also play a role in DCM susceptibility. Taken together, all of these limitations may have affected the results of this study.

## Abbreviations

CI: Confidence interval; DCM: Dilated cardiomyopathy; EDTA: Ethylenediaminetetraacetic acid; GWAS: Genome-wide association study; HSP: Heat shock protein; LD: Linkage disequilibrium; LV: Left ventricular; MALDI-TOF-MS: Matrix-assisted laser desorption/ionization time-of-flight mass spectrometry; MIZ-1: Myc-interacting protein 1; OR: odds ratio; PCR: Polymerase chain reaction; sHSP: small HSP; SNP: Single nucleotide polymorphism

## Competing interests

The authors declare no conflict of interest.

## Authors’ contributions

XL and RL performed all *in vitro* experimental studies and data acquisition, and contributed to the study conception, design, analysis, and data interpretation. XM, RJ, and HK collected serum samples, performed data analysis, and drafted the manuscript. WH and XW designed, set up, and monitored the study from which the serum samples were obtained. All authors read and approved the final manuscript.
